# Disruption of occludin function in polarized epithelial cells activates the extrinsic pathway of apoptosis leading to cell extrusion without loss of transepithelial resistance

**DOI:** 10.1186/1471-2121-10-85

**Published:** 2009-12-09

**Authors:** Neal E Beeman, Heidi K Baumgartner, Patricia G Webb, Jerome B Schaack, Margaret C Neville

**Affiliations:** 1 Department of Physiology and Biophysics, School of Medicine, University of Colorado Denver, Aurora, CO 80045, USA; 2 Department of Microbiology, School of Medicine, University of Colorado Denver, Aurora, CO 80045, USA; 3 Program in Molecular Biology, School of Medicine, University of Colorado Denver, Aurora, CO 80045, USA; 4 Department of Obstetrics and Gynecology, School of Medicine, University of Colorado Denver, Aurora, CO 80045, USA; 5 Dept of Pathology, Emory University, Whitehead Building, Rm 111, 615 Michael St., Atlanta, Georgia 30322, USA

## Abstract

**Background:**

Occludin is a tetraspanin protein normally localized to tight junctions. The protein interacts with a variety of pathogens including viruses and bacteria, an interaction that sometimes leads to its extrajunctional localization.

**Results:**

Here we report that treatment of mammary epithelial monolayers with a circularized peptide containing a four amino acid sequence found in the second extracellular loop of occludin, LHYH, leads to the appearance of extrajunctional occludin and activation of the extrinsic apoptotic pathway. At early times after peptide treatment endogenous occludin and the LYHY peptide were co-localized in extrajunctional patches, which were also shown to contain components of the death inducing signaling complex (DISC), caspases 8 and 3, the death receptor FAS and the adaptor molecule FADD. After this treatment occludin could be immunoprecipitated with FADD, confirming its interaction with the DISC. Extrusion after LYHY treatment was accomplished with no loss of epithelial resistance.

**Conclusion:**

These observations provide strong evidence that, following disruption, occludin forms a complex with the extrinsic death receptor leading to extrusion of apoptotic cells from the epithelial monolayer. They suggest that occludin has a protective as well as a barrier forming role in epithelia; pathogenic agents which utilize this protein as an entry point into the cell might set off an apoptotic reaction allowing extrusion of the infected cell before the pathogen can gain entry to the interstitial space.

## Background

Tight junctions, known as the *zonula occludens*, form an anastomosing network of protein and lipid strands that apically circumscribe every luminal cell of an epithelium. Classically, they form a continuous and selective barrier to paracellular solute flux and ionic current (the *gate *function) and help maintain the distinct lipid and protein composition of the apical and basolateral cellular membranes (the *fence *function). It is becoming increasingly clear that this structure is also the direct or indirect target of many pathogens including Hepatitis C virus [1], Coxsackie virus [2,3], *Clostridium perfringens *endotoxin [4], enteropathogenic *E. coli *[5,6], *Campylobacter jejuni *[7] and others [8] and that the tight junction protein occludin is often involved in host-pathogen interactions that result in infection.

**Figure 1:**
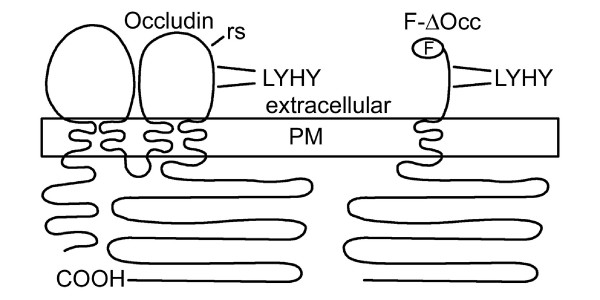
**Occludin and occludin disrupting molecules**. The general topology of the tetraspanin molecule occludin and the FLAG tagged truncated fragment used to make the truncated molecule, F-ΔOcc, are shown in diagrammatic format along with the location of an occludin disrupting peptide. COOH, C-terminus; PM, plasma membrane; F, FLAG epitope tag; rs, restriction site used to make truncated occludin; LYHY, conserved peptide sequence used in later experiments.

The tight junction is a complex and multifunctional structure consisting of integral membrane molecules, occludin, claudins and junction adhesion molecule. Occludin and the claudins are tetraspanin proteins with two extracellular loops and are considered to form the variable permeability barrier between the luminal and interstitial spaces separated by the epithelium. Tight junction plaque proteins such as ZO1, ZO2, and ZO3 [8] link the integral proteins to the actin cytoskeleton. They also interact with a diverse group of signaling molecules that connect tight junction function to paracellular permeability, cell division, cell polarity and tumorigenesis [8,9]. The focus of this paper, occludin, is a tetraspanin protein with four transmembrane domains, intracellular N and C termini and two extracellular loops (see Figure 1). Although occludin has been shown to be important in establishing and maintaining the physiological properties of the tight junction [10,11], and appears to be important for survival of cultured hepatocytes [12], the occludin-null mouse is viable and appears to have relatively minor alterations in epithelial function [13]. The claudins are now considered to play the major role in tight junction formation and adhesion [14]. Importantly for this work, interference with occludin either by overexpression of a truncated form [15] or with peptides that disrupt its cell recognition complex [16-18] has been shown to alter endothelial or epithelial permeability. More recently enteropathogenic *E. coli *have been shown to alter occludin localization in T84 epithelial cells and to bring about caspase mediated apoptosis [6]. Intriguingly, overexpression of occludin in a variety of tumor cells sensitized the cells to apoptosis inducing agents [19] and occludin negative clones showed reduced ability to extrude apoptotic cells from MDCK monolayers [20]. Thus there is conflicting evidence suggesting that occludin both enhances cell survival [12,20] and participates in reactions leading to apoptosis [6,19]. The work presented here suggests that occludin can have an important direct interaction with the extrinsic apoptotic pathway in the mammary epithelium.

Turnover of epithelial cells is a normal part of the differentiated function of the simple epithelia that line most mucosal surfaces; it has been best studied in the intestine and mammary gland. The intestinal monolayer is in a constant state of flux with cells originating in the crypt moving to the apex of the microvillus where they undergo apoptosis without a loss of epithelial integrity [21-23]. In the mammary gland a massive wave of apoptosis in the involuting gland leads to loss of 75% of the epithelial cells within 3 days [24,25] with no apparent loss of the barrier function until the process is well advanced ([24], Beeman and Neville, unpublished). In a landmark paper Rosenblatt [26] and her colleagues showed that isolated apoptotic cells are extruded from epithelial monolayers by the formation of an actinomyosin ring in neighboring cells; this ring gradually tightens around the extruding cell in such a manner that the neighboring cells maintain their intercellular contacts. Thus in epithelia the apoptotic process, called "cellular demolition" by Martin and his colleagues [27], occurs in a highly controlled fashion, removing apoptotic cells without a loss of epithelial integrity.

To determine whether changes in the tight junction could provide a link to extrusion and apoptosis of epithelial cells, we examined the effects of occludin disruption on mouse mammary epithelial cell monolayers with fully formed junctional complexes. We expressed truncated occludin [15] by transient transfection at low efficiencies. We also treated the monolayers with a peptide that mimics four amino acids in the second extracellular loop of occludin [16]. Both agents brought about an increase in non-junctional occludin that was associated with increased TUNEL staining, activation of caspases 8 and 3, and extrusion of cells from the monolayer with no changes in transepithelial resistance (TER). Most intriguing was our finding of occludin association with the death inducing signaling complex (DISC) [28], suggesting that occludin itself may act as a signaling molecule that activates the extrinsic pathway of programmed cell death.

## Results

### Expression of dominant-negative occludin using a plasmid construct

**Figure 2:**
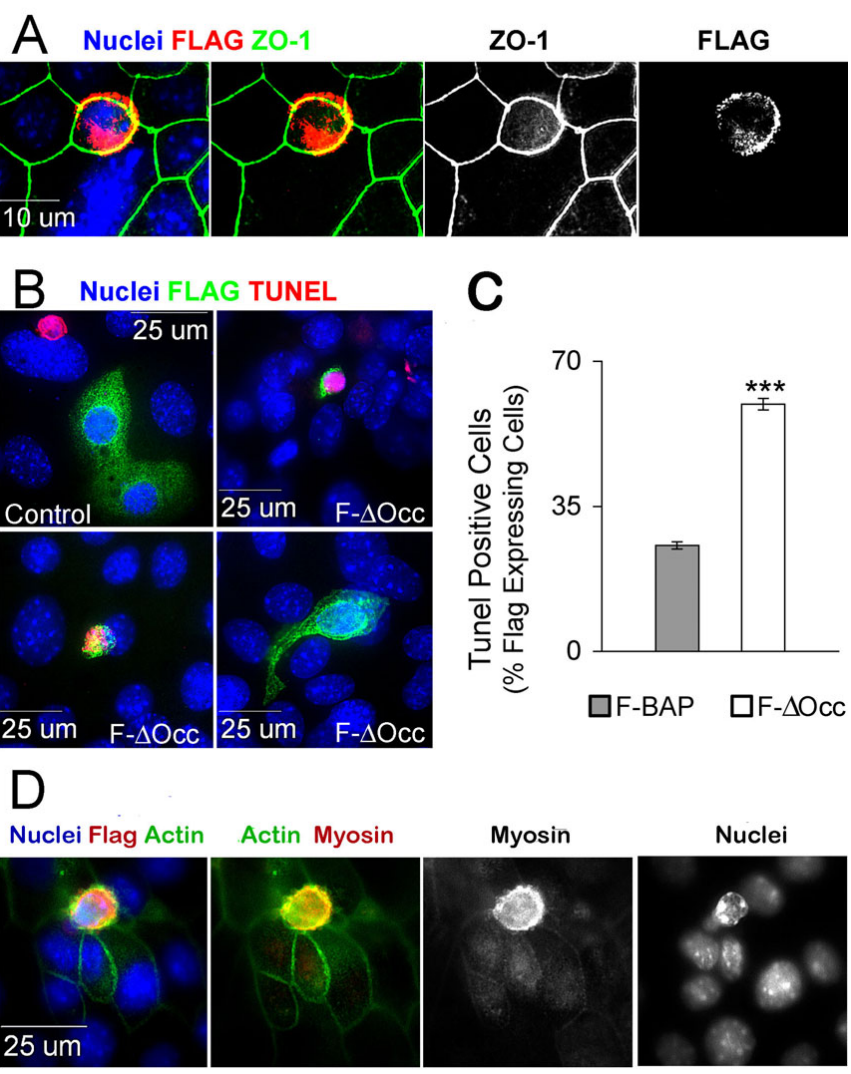
**Plasmid transfection of F-ΔOcc leads to extrusion and apoptosis**. A. CIT3 cells on glass slides transiently transfected with F-ΔOcc were fixed and stained 18 hr later. An antibody to ZO-1 outlines junctional complexes (green) and an antibody to FLAG (red) labels a transfected cell. Nuclei are stained with DAPI. Yellow in composite images represents overlap between ZO-1 and FLAG. B. CIT3 cells grown on glass slides were transiently transfected with FLAG-bacterial alkaline phosphatase as a control (upper left hand panel) or F-ΔOcc. After 48 hr monolayers were fixed and stained for TUNEL (red), FLAG (green), and nuclei (blue). A spontaneous TUNEL positive nucleus outside two F-BAP expressing cells (upper right panel) demonstrates that the TUNEL stain is working in this view. C. Transfected cells shown in B were identified by their expression of FLAG; the proportion of these cells showing TUNEL positivity was determined and plotted as a percentage of the FLAG stained cells. *** p < .0005. D. Cells transfected with F-ΔOcc as in B were visualized by staining for the FLAG epitope, actin, myosin, and nuclei as labeled.

In an initial experiment we examined the effects of occludin disruption by transiently transfecting CIT3 and Eph4 cells with plasmids expressing a flag-tagged truncated occludin, F-ΔOcc, as shown in Figure 1. Figure 2A shows FLAG and ZO1 co-staining of a single transfected CIT3 cell processed 18 hours after transfection. The FLAG tag was localized with ZO-1 at the tight junction as well as in intracellular puncta. The transfected cell is beginning to round up as is characteristic of the first step in extrusion [26].

Cells transfected with F-ΔOcc were stained for TUNEL and the FLAG tag 48 hours post transfection. Control cells were transfected with bacterial alkaline phosphatase bearing identical FLAG tag and secretory peptide sequences (F-BAP). Cells that expressed F-BAP maintained normal morphology (Figure 2B, control panel), whereas a variety of apoptotic morphologies were observed in the F-ΔOcc transfected cells. At 48 hr, 25% of control-transfected cells and 60% of F-ΔOcc-transfected cells were TUNEL positive (Figure 2C). Similar results were obtained with the Eph4 cell line (not shown). Close inspection of the stained cultures showed that the majority of F-ΔOcc-transfected cells were in various stages of extrusion and/or cellular demolition (Figure 2B&D). In a very occasional view (2B, lower right) an extrusion stalk could be seen extending apically from the monolayer. Nuclei in these extruding cells often were TUNEL negative.

Figure 2D shows an F-ΔOcc-transfected cell rising above the plane of the epithelium with a condensed but non-fragmented nucleus. A condensed ring of actin and myosin is visible at the level of the apical junction complex and below the plane of the nucleus in the extruding cell, suggesting that the mechanism of extrusion is similar to that observed by Rosenblatt et al [26]. Note that actin and myosin are not visible in the surrounding, non-apoptotic cells. Thus expression of a disrupted form of occludin in a mammary epithelial monolayer is associated with apoptosis and extrusion of the transfected cell.

### Treatment of polarized epithelial monolayers with an occludin disrupting peptide

**Figure 3:**
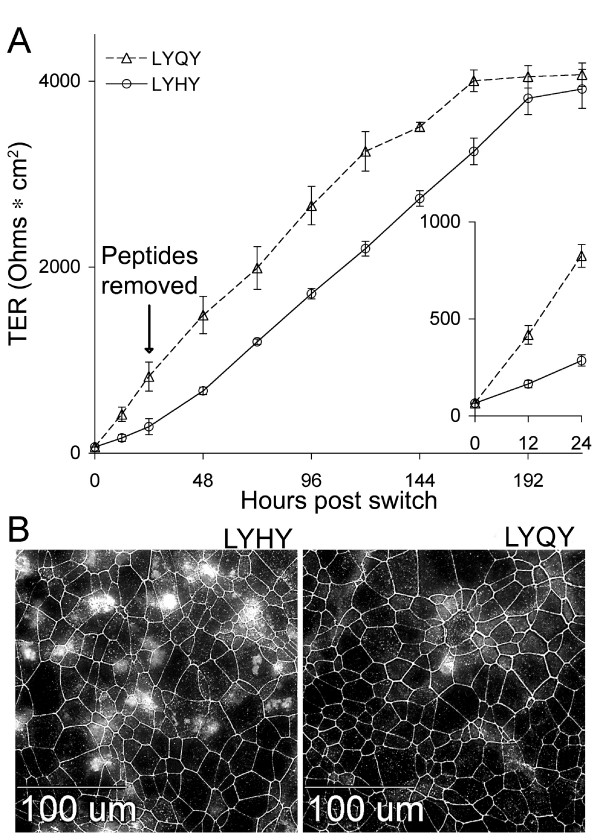
**Effects of an occludin second loop peptide (LYHY)**. A. Transepithelial resistance (TER) recovery following the calcium switch. EDTA was applied for 15 minutes to filter grown Eph4 monolayers that had achieved a TER of 1000 Ohms•cm2, reducing the TER to the baseline of 66 Ohms•cm2. LYHY or LYQY (350 μM) in growth medium containing normal Ca++ and Mg++ was placed above and below the filter supports. The TER was measured at 12 and 24 hours following peptide application (inset). Peptides were washed away 24 hrs following application and the TER measured every 24 hrs thereafter. B. Effect of peptides on occludin distribution in mature Eph4 cell monolayers. EPH4 cells on glass slides were treated with 350 μM LYHY or LYQY for 8 hours then fixed and stained with an anti-occludin antibody. Note the frequent areas of non-junctional occludin in the LYHY treated cells compared to the control LYQY peptide.

The next question was whether disruption of occludin by another means would produce a similar result. We utilized a peptide previously reported to disrupt tight junctions [16]. This peptide contained the LYHY sequence from the second extracellular loop of mouse occludin circularized for stability by oxidation of flanking cysteine residues. To confirm that this peptide disrupted the tight junction, we performed a "calcium switch" experiment on filter-grown Eph4 cells with an initial TER of 1000 Ohms•cm^2^, removing extracellular Ca^++^and Mg^++ ^to disrupt intercellular junctions. After 15 minutes in the divalent cation depleted-medium TER was reduced to zero. Cells were then switched back to normal medium containing the LYHY or control LYQY peptide (350 μM). The control peptide (LYQY) has a one amino acid substitution of the occludin peptide and was similarly circularized. Peptides were removed after 24 hours. Control peptide-treated cells recovered the initial TER by 48 hours and the TER continued to climb, reaching 4000 Ohms•cm^2 ^(Figure 3A). The occludin peptide severely retarded TER recovery during the 24 hours it was present in the medium (see inset), although once removed, the TER began to climb in parallel with the control.

Treatment of a confluent monolayer of Eph4 cells with 350 μM LYHY peptide for 8 hours (Figure 3B) led to the appearance of large numbers of cells with irregular non-junctional occludin distributed in patches. Higher concentrations of peptide produced severe disruption of the monolayer (not shown). Because we were interested in examining effects on single cells, unless noted, experiments were conducted with 350 μM peptide. Only an occasional cell showing a non-junctional distribution of occludin was observed in monolayers treated with the control peptide. Similar results were obtained with CIT3 and MDCK cells (data not shown). The next question was whether this disruption of non-junctional occludin led to apoptosis.

### Occludin peptide treatment increased apoptosis in polarized epithelial cell monolayers

**Figure 4:**
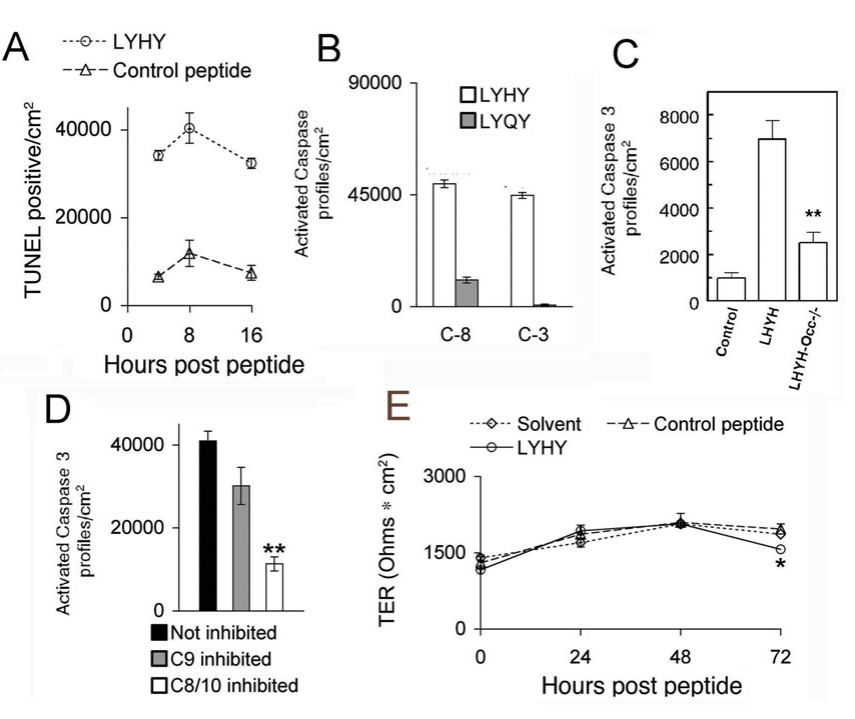
**LYHY causes apoptosis with no change in TER**. A. Eph4 cells were grown on glass slides and treated with LYHY or control LYQY peptide for 4, 8, and 12 hours. Cells were TUNEL stained and counted (see Additional File 1A). B. Eph4 cells were peptide treated and stained live with sulphorhodamine-DEVD-fluoromethyl ketone for activated caspase 3 (C-3) and with carboxyfluorescein-LETD-fluoromethyl ketone for caspase 8 (C-8) (see Additional File 1B), fixed, photographed and counted. C. Primary cultures from the mammary gland of a control mouse and an occludin null mouse were treated with the LYHY peptide and stained with an antibody for cleaved caspase 3. Control is no peptide. **p < 0.01 compared to LHYH treated Occ^++ ^cells, middle bar. D. Mature Eph4 monolayers on glass slides were pre-treated for 2 hours with a peptide inhibitor of caspase 8/10 or caspase 9, or solvent only (Not inhibited). Cells were then treated with LYHY peptide for 2 hours and the number of cells showing activated caspase 3 counted. E. Eph4 cells were grown on filters until the TER was ~1400 (Ohms•cm2), then treated with solvent, LYHY, or control LYQY peptide at 350 μM in both top and bottom chambers. Peptides were replenished daily, after recordings. Asterisk indicates a statistically significant fall in TER at 72 hours (p < .05) relative to control peptide. Results of three wells averaged.

We treated Eph4 monolayers with the LYHY peptide for 12 hours then examined TUNEL staining by immunohistochemistry (Additional File 1A). Quantitation of TUNEL stained cells showed that large numbers of apoptotic cells were present at 4 hours compared to cultures treated with the control LYQY (Figure 4A). To be sure that occludin was the target of this reaction, we plated primary cells derived from the mammary gland of a pregnant occludin knock-out mouse obtained from M. Furuse [13] and obtained monolayer cultures. Control cells were obtained from a wild type female. Both were treated with the LYHY peptide for 16 hours and stained with an antibody to the effector caspase 3. Increased apoptosis was observed in cultures from the wild type but not the occludin-null mice, providing strong evidence that occludin is necessary for the apoptotic reaction.

The next question was whether we were dealing with the extrinsic or intrinsic pathway of apoptosis. Both pathways utilize an "initiator" caspase to activate the effector caspase, caspase 3; however, both the site of the initial reaction and the initiator caspase differ between the two. In the intrinsic pathway cell stress or damage leads to release of cytochrome C from the mitochondria [27]. The cytochrome C seeds a remarkable "apoptosome" assembly leading to activation of the initiator caspase 9 which in turn activates caspase 3. The external apoptotic pathway starts with activation of transmembrane death receptors such as FAS, TRAIL and others. Activation of these receptors leads to binding of an adaptor protein FADD (Fas-associated death domain protein), which in turn binds and aggregates caspase 8, promoting its autoactivation. Activated caspase 8 in turn cleaves and activates pro-caspase 3 leading to the full apoptotic reaction. We tested the hypothesis that the pathway activated by the occludin peptide was the extrinsic pathway in two ways. We first examined activation of the initiator caspase 8 and the effector caspase 3, costaining live cells with labeled peptides specific for the active sites of the two caspases. Interestingly slightly more cells reacted with the caspase 8 peptide than the caspase 3 peptide (Figure 4B and Additional File 1B). Further, cells could be observed with activated caspase 8 alone but never with activated caspase 3 in the absence of caspase 8 (Additional File 1B). These results suggested that LHYH activates the death receptor pathway and that caspase 8 is the initiating caspase. These caspases were only slightly activated by the control peptide LYQY (Figure 4B).

**Figure 5:**
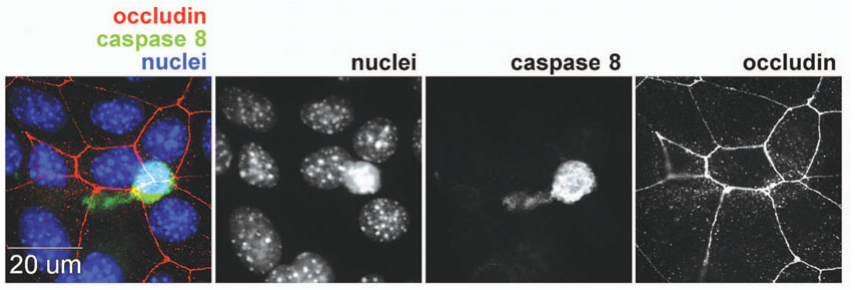
**Extrusion of a caspase stained cell after treatment with LYHY**. Mature Eph4 monolayers grown on glass slides were treated with LYHY peptide at 350 μM for 6 hours. They were stained live during the 6th hour of treatment with carboxyfluorescein-LETD-fluoromethyl ketone for activated caspase 8 as described in Materials and Methods. After fixation monolayers were stained for occludin and nuclei. An extruding cell stained for caspase 8 shows a narrow extrusion "tail" and has a slightly pyknotic nucleus. Surrounding cells have closed the gap around the extruding cell as shown by occludin staining. Occludin is no longer visible in the extruding cell. Black and while images show separate channels for nuclei, caspase 8 and occludin staining. The merged colored image is on the left.

To provide a second, more rigorous test of the hypothesis that caspase 8 is the initiating caspase we measured the number of cells showing activation of the downstream effector caspase 3 during inhibition of caspase 8/10 [28]. Mature monolayers were incubated with inhibitors of caspase 8 and 10 (Z-IETD-FMK and Z-AEVD-FMK respectively), no inhibitor, or an inhibitor (Z-LEHD-FMK) of caspase 9, the initiator caspase of the mitochondrial apoptotic pathway. Monolayers were then treated with cyclic LYHY peptide and stained for activated caspase 3 (Figure 5C). The number of cells staining for activated caspase 3 during caspase 8/10 inhibition decreased 75%. Caspase 9 inhibition produced a reduction of 25% or less. Similar results were obtained when only the caspase 8 inhibitor (Z-IETD-FMK) was used in this experiment, as expected, since mouse cells do not express caspase 10 (data not shown). Our findings provide strong evidence that internalization of the LYHY peptide with non-junctional occludin leads to activation of the extrinsic pathway of apoptosis. With this concentration of peptide cells are extruded from the monolayer with no loss of epithelial resistance (Figure 4E).

To examine the mechanism of loss of apoptotic cells, we applied the LHYH peptide for 6 hours, adding the live cell probe for activated caspase 8 during the last hour. Careful microscopic examination revealed occasional caspase 8 staining cells being extruded from the monolayer, complete with extrusion stalk (Figure 5) consistent with the extrusion mechanism shown earlier with F-ΔOcc.

### Non-junctional occludin interacts with the Death-Inducing Signaling Complex (DISC)

**Figure 6:**
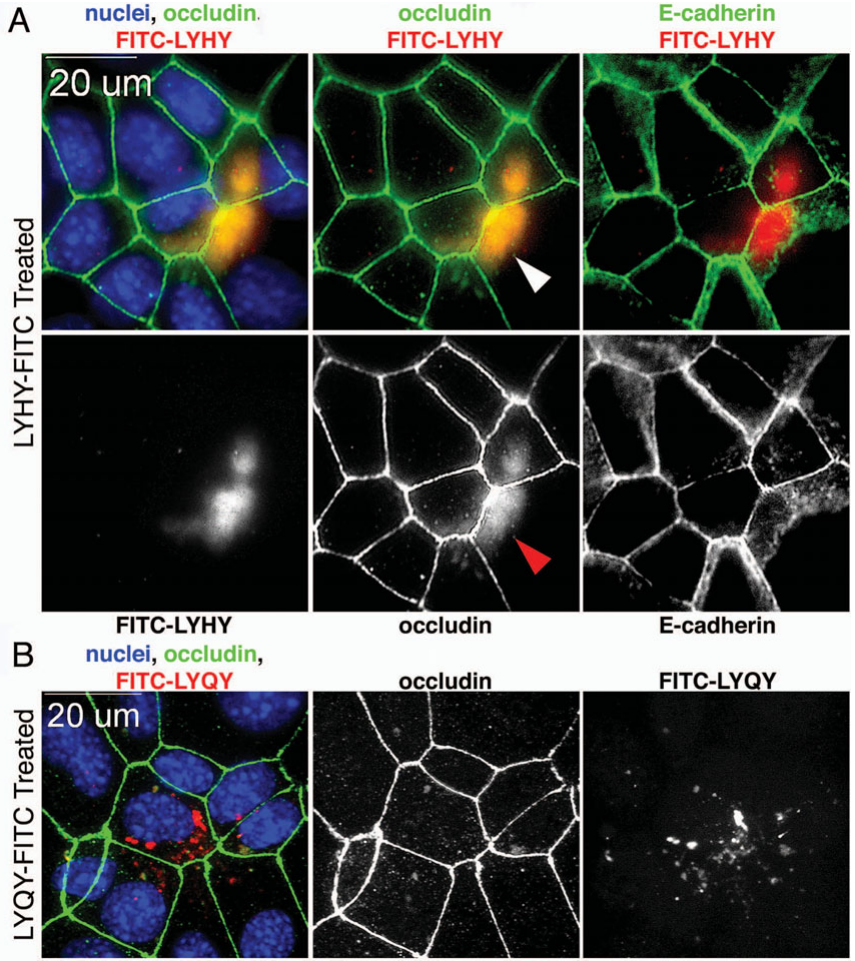
**Peptide localization in EPH4 cells**. EPH4 cells grown on glass slides were treated with FITC labeled LYHY (A) or LYQY (B) peptides (350 μM) for 6 hr (pseudocolored red). After fixation, cells were stained with antibodies against occludin (green pseudocolor) and E-cadherin (green pseudocolor) and with DAPI for nuclei (blue). LYHY accumulated in regions enriched in non-junctional occludin, denoted by arrowhead. LYQY did not alter occludin distribution although it appeared to localize in the cytoplasm of some cells. E-cadherin distribution was not altered by LYHY.

The question now is "What is the pathway by which the peptide leads to activation of the extrinsic apoptotic pathway?" We first asked where the LYHY peptide localized by treating the cell with a FITC-labeled cyclic LYHY peptide. We found that this peptide did indeed colocalize with non-junctional occludin (Figure 6A) in contrast to the control FITC-labeled LYQY peptide, which neither caused internalization of occludin nor became enriched in regions of displaced occludin (Figure 6B). E-cadherin distribution was not changed by the peptide (Figure 6A) indicating that the adherens junction was not disrupted. This experiment provides evidence that the LYHY peptide interacts with occludin, but does not provide information about the nature of the "occludin patches" or their localization with respect to the polarity of the epithelial cells. The next experiments were designed to examine these issues.

### Colocalization of occludin and caspases with components of the death receptor pathway

**Figure 7:**
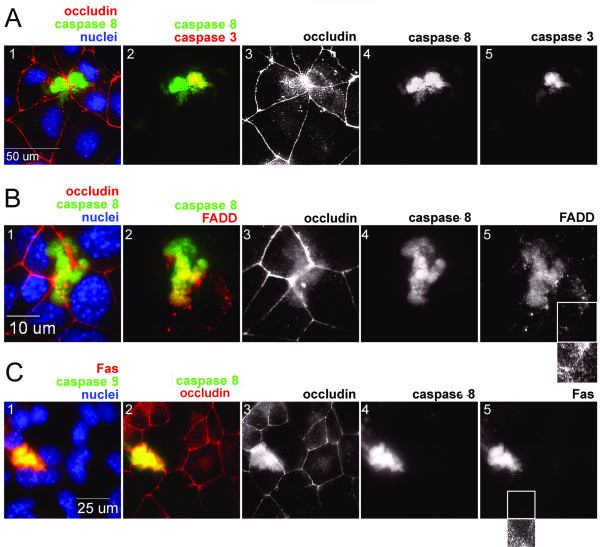
**Localization of caspase 8 with displaced occludin and DISC proteins after LYHY treatment of Eph4 monolayers on glass slides**. A. Cells were treated with LYHY at 350 μM for 30 minutes and stained live during peptide treatment for activated caspase 8 (green, A1, 2) and activated caspase 3 (Red, A2). Cells were then fixed and stained for occludin (red pseudocolor, A1) and nuclei (Blue). Activated caspase 3 co-localizes with disrupted occludin and with activated caspase 8 in the right hand cell only. B. EPH4 cells on glass slides were treated for 2 hr with LYHY then stained live for activated caspase 8 (green B1, B2). After fixation monolayers were stained for occludin (pseudocolored red, B1), the DISC adapter protein FADD (red, B2) and nuclei (blue). The smaller panel under B5 shows an image that has been exposed for a longer time, and suggests that FADD is present at lower intensity in regions free of non-junctional occludin. C. EPH4 cells were treated with LYHY and stained for activated caspase 8 (green C1, C2; C4) and occludin as well as the death receptor Fas (red, C1 and white C5), and nuclei (blue). The smaller panel under C5 shows a more highly contrasted view of the region within the white box of the Fas panel. Like FADD, low levels of Fas appear to be present in cellular regions free of non-junctional occludin.

Because we had evidence that treatment with peptide leads rapidly to apoptosis, we stained Eph4 cells live for activated caspases 8 and 3 after 30 minutes of treatment with LHYH. Cells were then fixed and stained with an antibody to occludin (Figure 7A). An intense patch of activated caspase 8 substantially colocalized with non-junctional occludin in two adjacent cells. One of these cells also showed activated caspase 3, colocalized with caspase 8. If occludin and these caspases are localized to the DISC, components of the death receptor should also be present in these regions. In fact, immunostaining showed that Fas and FADD are localized to the same regions as non-junctional occludin and caspase 8 (Figure 7B &7C). Thus the DISC appears to contain Fas, FADD, and caspases 3 and 8 and occludin, suggesting death receptor activation by non-junctional occludin.

**Figure 8:**
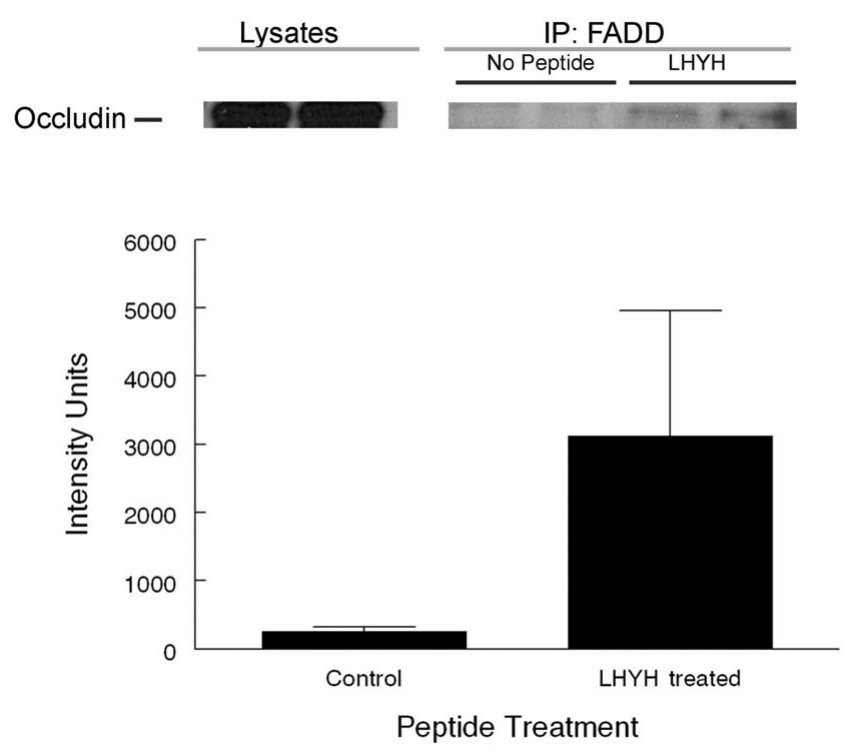
**Co-immunoprecipitation of occludin with FADD after treatment with LYHY**. Eph4 cells were treated with 800 μM LHYH for 5 hrs or left untreated after medium change. Cells were lysed and proteins immunoprecipitated with a mouse anti-FADD antibody using protein A/G agarose beads and blotted with a rabbit anti-occludin antibody. A. Western blot for occludin. Lysates of cells that were immunoprecipitated with an anti-FADD antibody. IP-FADD: Two left lanes show IP of lysates from untreated EPH4 cultures and two right hand lanes show IP of lysates from LYHY treated EPH4 cultures. A very small proportion of the total cellular occludin was associated with FADD. B. Quantitation of occludin bands from 3 control and treated cultures. The intensity of stain was variable but higher in the immunoprecipitated fractions from LHYH treated cells (P < 0.1). Similar results were obtained from 2 comparable experiments.

If occludin forms a true complex with the disc we should be able to immunoprecipitate it with FADD, a component of the DISC. In Figure 8A a band corresponding to occludin can be seen after immunoprecipitation of FADD from LYHY treated cells. This figure also shows that the proportion of occludin that can be immunoprecipitated is small and somewhat variable, Figure 8B. Similar results were obtained in 3 independent experiments.

### Localization of the DISC aggregate

**Figure 9:**
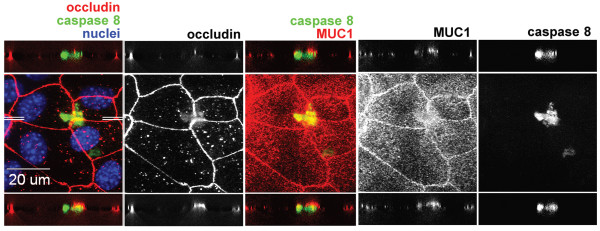
**Cellular localization of activated caspase 8 and non-junctional occludin**. Cells were grown and stained as in Figure 7A except that the apical plasma membrane marker MUC1 (red, third panel, white 4^th ^panel) was stained in place of caspase 3. Two sets of white lines in the first panel show the plane of two slightly different digital XZ-sections projected above and below the central XY image in this composite image.

The next question is how the localization of the DISC relates to the polarity of the cell. Using digital confocal microscopy we examined a Z-stack of images from the apex to the basal surface of the cell. To mark the apical surface cells were co-stained with an antibody to MUC1, a mucin localized exclusively to the apical surface of the cell (Figure 9). The middle images show the XY plane. The ZX plane was calculated at two different positions of Y, as shown by the white lines in the middle left image, and the ZX images are depicted above and below the XY images. In these images activated caspase 8 appeared to be localized just basal to MUC1 and the main body of occludin staining. However, there is a fuzzy projection of occludin into the region of caspase 8 staining as shown by the yellow band separating the red occludin and green caspase 8 bands in the lower left hand image. Thus the DISC is located apically and the occludin associated with the DISC appears to be in the membrane suggesting that occludin moves through the membrane to activate the death receptor.

## Discussion

To examine the potential role of tight junction disruption in the controlled demolition of the epithelial cell, we used two previously published occludin-disrupting tools in three epithelial cell lines (Eph4, CIT3, and MDCK) as well as primary cultures of the mammary epithelium. We found that both a truncated occludin construct and a peptide identical to 4 amino acids in the second extracellular loop of occludin increased non-junctional occludin, increased activation of caspases 8 and 3, and brought about TUNEL staining in treated cells. Cells with these signs of apoptosis were extruded from the monolayer with no change in TER. There was no increased apoptosis in occludin null cells exposed to the peptide. At early times non-junctional occludin was localized in the DISC with activated caspases 8 and 3 as well as with the death receptor FAS protein and the adaptor protein FADD. It could be co-immunoprecipitated with FADD. These findings suggest that disruption of occludin leads to its displacement from the tight junction to the DISC, initiating extrusion and apoptosis of the affected cell by the Type I extrinsic pathway [29].

### Expression of truncated occludin

The F-ΔOcc construct had been previously used as a dominant negative occludin to transfect cultured salivary cells [15]. These cells showed normal distribution of endogenous occludin, ZO-1, and JAM. However, tracer flux was increased and TER decreased. This experiment differed from ours in that the salivary cells were stably transfected and constitutively expressing, surviving cells were selected. We used low concentrations of plasmid to obtain transient transfection of sparsely located cells, which then underwent apoptosis and left the monolayer. It would be of considerable interest to determine how the stably transfected monolayers were able to adapt to expression of the mutant construct.

### Peptides mimicking other occludin sequences

Tight junction disruption via peptides containing sequences in the second extracellular loop of occludin has been previously reported [17,18,30,31]. When intact EPH4 cell monolayers were treated with a 44 amino acid peptide comprising the entire second extracellular loop of chicken occludin, isolated patches of cells throughout the monolayer showed a punctate, intracellular, non-junctional distribution of occludin [31]. These peptide-treated cells maintained a TER above 3000 Ohms·cm^2^, about 50% of that of controls. In another laboratory a peptide mimicking a 22 amino acid sequence in the second extracellular loop impeded recovery of TER and caused internalization of several tight junction transmembrane proteins following the calcium switch in human intestinal epithelial cells [17]. Similar results were obtained with a rat 19 amino acid second extracellular loop mimic in a rat Sertoli cell line [30]. In these latter two studies the peptide ended two amino acids upstream of the LYHY sequence suggesting that regions comprising peptides other than LYHY are also involved in homophilic interactions of occludin.

### Extrusion and TER

The finding that a large increase in apoptosis, which occurred in all studies, did not decrease the trans-epithelial resistance suggests that loss of single epithelial cells proceeds by an orderly biological process that maintains rather than disrupts the epithelial barrier. Similarly, cultured monolayers of intestinal epithelial cells were able to maintain 50% of basal TER when treated with a Fas crosslinking antibody that led to the loss of half of the cells in the culture in only 24 hours [23]. Because we observed an actomyosin "purse string" around extruding cells in our study (Figure 2D), the mechanism of extrusion first elucidated by Rosenblatt and colleagues [26] is likely used.

### Is displaced junctional occludin acting as a signaling molecule?

It has been known for some time that viral and bacterial pathogens can enter cells utilizing occludin and other elements of the tight junction [1-6,8]. In fact, Greber and Gustaldelli [3] have called the tight junction the "Achille's heel of epithelial cells in pathogen infection". Our studies raise the very important question of whether signaling through disrupted occludin could provide a significant host defense against, at least moderate concentrations of, pathogens by producing controlled extrusion and apoptosis of affected cells. A recent study of the induction of epithelial cell apoptosis by enteropathogenic *E. coli *(EPEC) is consistent with such a mechanism [6]. EPEC infection led to the cleavage of occludin and ZO-1 and the mis-localization of these molecules. Subsequently an increase in apoptosis markers was observed. We only observed full length occludin in FADD pulldowns, suggesting that the peptide in some way releases full length occludin from the tight junction freeing it to interact with apical membrane FAS and FADD prior to any cleavage. This reaction contrasts with the finding that occludin prevents apoptosis in cultures of hepatic cells [12]. It is possible that occludin can both stabilize junctional complexes and signal to a cell that its junctional complex has been perturbed by a pathogenic agent. If so, occludin is quite a versatile molecule.

Schneeberger has previously suggested that occludin might serve as a signaling molecule, although in a different context [20]. While the results of our studies are consistent with a role for non-junctional occludin as a signaling molecule, it is clear that many other signaling molecules are also associated with tight junctions [3]. Any one of a number of these could transmit a signal from a disrupted tight junction to the extrinsic apoptotic pathway. An intriguing possibility is that the Akt antagonist, lipid phosphatase PTEN (phosphatase and tensin homolog deleted from chromosome 10) links loss of occludin function to apoptosis. Several reports have demonstrated that the PTEN binds to the tight junction associated MAGI-2 [32], PAR [33,34], and DLG [32] proteins and PTEN binding to the PDZ domain of Magi-2 promoted its stability [35]. Moreover, PTEN plays a role in activation of the death receptor pathway of apoptosis under various conditions [36-38]. Our preliminary results show that PTEN is associated with the tight junction in Eph4 cells (data not shown). It will be of interest to determine whether it plays a role in linking occludin disruption to death receptor mediated apoptosis.

### Future directions

The results of our experiments link disruption of the tight junction protein occludin to stimulation of apoptosis via the extrinsic pathway and provide a potential defense mechanism for ridding the epithelium of cells exposed to pathogens. They also raise questions for further experimentation: For example, does the apoptotic process involved meet criteria for Type I death receptor activation other than the recruitment of large amounts of caspase 8 to the DISC [29]? Interestingly, Nusrat and her colleagues have proposed that the tight junctional components reside in lipid raft-like complexes [39]. The diffuse nature of the occludin stain in Figures 6, 7 and 9 leads us to ask whether portions of these rafts might detach from the junction and fuse with the DISC, which has also been shown to localize to lipid rafts [40].

## Conclusion

We have found that a small cyclized peptide LYHY disrupts occludin localization at the tight junction and activates the extrinsic pathway of programmed cell death. At early times occludin is localized at the death inducing signaling complex and can be immunoprecipitated with FADD, a member of this complex. These observations suggest that occludin has a protective as well as a barrier forming role in epithelia; pathogenic agents which utilize this protein as an entry point into the cell might set off an apoptotic reaction allowing extrusion of the infected cell before the pathogen can gain entry to the interstitial space.

## Methods

### Antibodies

Anti occludin, Zymed^® ^clone OCOC-3F10; anti Fas, BD Biosciences clone 13; anti ZO-1, Chemicon^® ^MAB1520; anti FADD, USBiological clone 12E7; anti MUC1, Abcam inc. clone EP1024Y; anti-caspase 3, Cell Signaling Technology^® ^(8G10); anti-caspase 8, Axxora^® ^(1G12).

### Mice

A breeding colony of occludin null homozygous mice is maintained in our animal facility. To derive occludin-null cells male and female hemizygotes are cross-bred and female homozygotes are selected and bred. These mice can lactate, although lactation performance is inconsistent (Monks, Webb and Neville, unpublished). For the current experiments, mice whose pregnancies were timed by the appearance of a vaginal plug, were sacrificed at day 15 of pregnancy and the 4^th ^and 5^th ^mammary glands excised and collagenase treated as described previously to obtain isolated epithelial organoids [41]. These organoids are plated on FBS treated Lab Tek II, CC2 glass chamber slides (Nunc) where they form nearly confluent monolayers with well formed junctions as indicated by ZO1 staining within 3 to 6 days of culture (Wilson and Neville, data not shown). Organoids from wild type mice are handled similarly. These procedures have been approved by the Institutional Animal Care and Use Committee of the Anschutz Medical Campus of the University of Colorado Denver.

### Cells and Cell culture

EpH4 mammary epithelial cells [42] were grown in DMEM (GibcoBRL, Grand Island, NY) with 10 mM HEPES (Sigma-Aldrich) and subcultured every 3-4 days [41]. CIT3 mouse mammary epithelial cells were grown as described [43] in DMEM with Ham's F12 (50:50) supplemented with 2% heat-inactivated fetal bovine serum (FBS), 5 ng/ml epidermal growth factor (EGF), 10 mg/ml insulin, 100 U/ml penicillin and 100 mg/ml streptomycin. To differentiate CIT3 cells, the growth medium was modified by removal of EGF and addition of 3 mg/ml each of ovine prolactin (National Hormone and Pituitary Program, Rockville, MD) and hydrocortisone (Sigma Chemical Co. St. Louis, MO). MDCK cells were grown in MEM with 2 mM L-glutamine and Earle's BSS adjusted to contain 1.5 g/L sodium bicarbonate, 0.1 mM non-essential amino acids, 1.0 mM sodium pyruvate, and 10% FBS [44]. Cultures were maintained at 37°C in 5% CO_2 _in air. For primary cultures mammary epithelial cells from 15 day pregnant mice were isolated as described above.

For immunohistochemistry and measurement of transepithelial resistance, cells were trypsinized from polycarbonate flasks, plated at 1:2 and grown for 7 days, then plated at 2× confluent density on FBS treated Lab Tek II, CC2 glass chamber slides (Nunc) or Transwell^® ^filters (Product #3413) as stated in figure legends. Cells were grown 3 days then, for CIT3 cells, switched to medium containing hormones for 2 days prior to the beginning of experiments.

### Expression constructs and transient transfection

Mouse occludin ATCC #MGC-5797 was cut with BsaA1 in the second extracellular loop and Bcl1 in the 5' UTR and inserted in pFLAG-CMV-1 (Sigma-Aldrich), placing it downstream of a secretory signal peptide and N-terminal FLAG epitope tag (F-ΔOcc). As a control vector pFLAG-CMV-1-BAP, which encodes secretory, N-terminal FLAG tagged, bacterial alkaline phosphatase, was obtained from Sigma-Alrich. F-ΔOcc or pFLAG-CMV-1-BAP was transfected into cells using FuGENE^® ^HD (Roche) according to the manufacturer's instructions. Cells were plated in the morning at confluent density, transfected in the evening, and transfection medium was replaced with differentiation medium the following morning.

### Peptide synthesis

Synthesis of linear CLYHYC was accomplished using Fmoc chemistry. Assembly of side-chain protected N-α-Fmoc amino acids (AAs) was carried out on a resin support. Acylation reactions were carried out for 2 hours using 3-fold excess of Fmoc-AAs activated with N, N'-diisopropylcarbodiimide (DIC) in the presence of N-hydroxybenzotriazole (HOBt). The N-terminal Fmoc group was removed with 20% piperidine in N,N-dimethylformamide. Linear peptide was cleaved from the fully dried peptide-bound resin with freshly prepared TFA solution (TFA 94%, water 2.5%, ethanedithiol 2.5%, triisopropylsilane 1%) for 2.5 hours under nitrogen atmosphere and then cyclized with 5% iodine in methanol and purified to 95% pure cyclic H-CLYHYC-OH by reverse phase HPLC. Peptide mass was confirmed by MALDI-TOF mass spectrometry (expected [M+H]^+ ^799.9, found 799.8). Cyclic H-CLYQYC-OH was made using the same process except that H was replaced by Q. Peptide mass was confirmed by mass spectrometry (expected [M+H]^+ ^790.9, found 790.6).

### Activated caspase stains

Cells were live stained with Image-iT^®^LIVE Green Caspase 8 Detection Kit (Molecular Probes) and/or Image-iT^®^LIVE Red Caspase 3 and 7 Detection Kit (Molecular Probes). Stains were diluted into differentiation medium containing LYHY or LYQY. After 1 hr at 37° cells were rinsed in 4°C differentiation medium, and processed for immunofluorescence.

### Caspase 9 and caspase 8/10 inhibition

Caspase 9 inhibitor (Z-LEHD-FMK Cat.#FMK008), 100 μM, caspase 8 inhibitor (Z-IETD-FMK Cat.#FMK007), 50 μM, or caspase 10 inhibitor (Z-AEVD-FMK Cat.#FMK009) (R&D systems), 50 μM, was dissolved in medium and incubated with cells for 2 h. LYHY peptide was then applied at 350 μM with inhibitors for 2 hours total. Caspase 3 staining was performed during the last hour of LYHY treatment as described above.

### Immunofluorescence

Cells were fixed in 2% paraformaldehyde, then permeabilized and blocked in 3% BSA in 0.1% Triton X-100 TBS. Antibodies were diluted into this solution and incubated with cells overnight at 4°C. In all cases secondary antibodies were goat anti host IgG, highly cross-absorbed, with appropriate fluors obtained from Molecular Probes.

### TUNEL Staining

TUNEL staining was performed using the Roche In Situ Cell Death Detection Kit, TMR red. Cells were fixed in 2% paraformaldehyde and permeabilized in 1% sodium citrate (tri-sodium salt) containing 0.1% TX-100. Staining was performed as per manufacturer's instructions.

### Fluorescence imaging

Images were collected, processed and analyzed using SlideBook software (Intelligent Imaging Innovations, Inc.) on a Nikon Diaphot TMD microscope equipped for fluorescence with a Xenon lamp and filter wheels (Sutter Instruments), fluorescent filters (Chroma), cooled CCD camera (Cooke) and stepper motor (Intelligent Imaging Innovations, Inc.). Adjacent z-sections were collected and deconvolved using a nearest neighbor algorithm. Multi-fluor images were merged and renormalized.

### Immunoprecipitation

Cells were washed with ice cold PBS before 0.5 ml of lysis buffer (30 mM Tris HCl pH 7.4, 150 mM NaCl, 1% Triton X-100, 10% glycerol, 2 mM EDTA, 5.7 μl/ml 100 mM PMSF, 20 μl/ml Roche Complete EDTA-Free Inhibitor Cocktail, and 1 μl/ml 1 M DTT) was added to the culture plate. Cells were scraped from the plate and placed in a microfuge tube. Cells in lysis buffer were constantly agitated for 20 minutes at room temperature and then spun at 13, 900 rpm for 20 minutes. The supernatant was collected in a fresh tube and stored at 4°C.

Lysates containing 500 μg protein were incubated with 10 μg mouse anti-FADD (BD Biosciences) antibody for 2.5 hours at room temperature. Protein A/G Agarose beads (20 μl, Santa Cruz) were added to the lysate/antibody mixture for 1 hour before being washed 3 times in lysis buffer, followed by a wash in high salt lysis buffer (regular lysis buffer with 500 mM NaCl), followed by a final wash in regular lysis buffer. Bead/antibody/FADD complex was resuspended in 50 μl 2 × Llaemmili buffer and boiled for 5 minutes. The sample was then spun at 2, 500 rpm for 5 minutes and the supernatant was collected in a fresh tube and stored at 4°C. Samples (30 μg original cell lysate, 25 μl IP product) were loaded on a 12% acrylamide gel and run at 100 Volts for about 1.5 hours. The gel was transferred onto PVDF and treated with rabbit anti-occludin antibody (1:2000, Santa Cruz) at 4°C overnight. The membrane was then treated with a donkey anti-rabbit IgG, Horseradish Peroxidase linked antibody (GE Healthcare).

## Abbreviations

Ad-F-ΔOcc; N-terminally FLAG tagged: N-terminally truncated mouse occludin mutant; BAP: bacterial alkaline phosphatase; DISC: Death inducing signaling complex; EPEC: enteropathogenic *Escherichia coli*; GFP: green fluorescent protein; TER: transepithelial resistance; TUNEL: Terminal deoxynucleotidyl transferase dUTP nick end labeling.

## Authors' contributions

NEB conceived and conducted all the experiments presented with the exception of those from the occludin null mouse and the immunoprecipitation. MCN and JBS supervised the work and provided continuous guidance. HKB and PGW worked out and performed the immunoprecipitation and the experiments with primary cultures from the occludin-null mouse. MCN formulated the final drafts of the paper. All authors have read and approved the final manuscript.

## Supplementary Material

Additional file 1 **Effect of LYHY on TUNEL reactivity and Caspase activation (figure)**. A. Eph4 cells were grown on glass slides and treated with LYHY or control LYQY peptide for 12 hours. Cells were TUNEL stained. B. Mature Eph4 monolayers on glass slides were treated with the LYHY peptide (panels B1-3) or with LYQY (panels B4-6) at 350 μM for 2 hours and stained live for activated caspase 8 (green; carboxyfluorescein-LETD-fluoromethyl ketone) and caspase3 (red; sulphorhodamine-DEVD-fluoromethyl ketone). Yellow regions in panels 1 and 4 represent colocalization. The arrows in panels 1 - 3 indicate regions of caspase 8 activation with no apparent caspase 3 activation. Click here for file
