# Supplementary figures and images for: Disruption of occludin function in polarized epithelial cells activates the extrinsic pathway of apoptosis leading to cell extrusion without loss of transepithelial resistance
Source: BMC Cell Biol. 2009 Dec 9;10:85. doi: 10.1186/1471-2121-10-85 (PMC2796999; doi:10.1186/1471-2121-10-85)

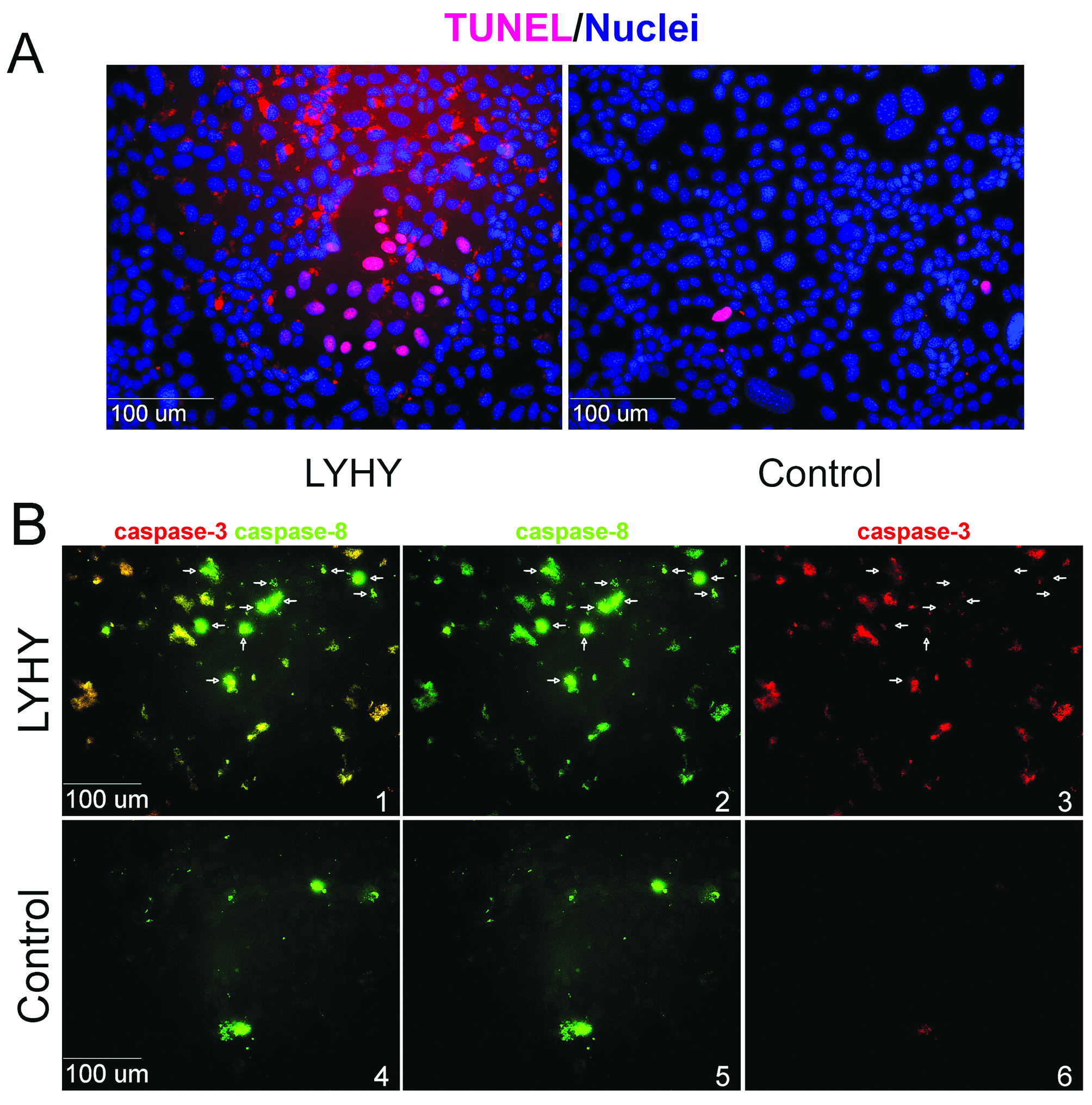

Supplement: Additional file 1 — Effect of LYHY on TUNEL reactivity and Caspase activation (figure). A. Eph4 cells were grown on glass slides and treated with LYHY or control LYQY peptide for 12 hours. Cells were TUNEL stained. B. Mature Eph4 monolayers on glass slides were treated with the LYHY peptide (panels B1-3) or with LYQY (panels B4-6) at 350 μM for 2 hours and stained live for activated caspase 8 (green; carboxyfluorescein-LETD-fluoromethyl ketone) and caspase3 (red; sulphorhodamine-DEVD-fluoromethyl ketone). Yellow regions in panels 1 and 4 represent colocalization. The arrows in panels 1 - 3 indicate regions of caspase 8 activation with no apparent caspase 3 activation. [file 1471-2121-10-85-S1.TIFF]
